# High Latitude Ionospheric Gradient Observation Results from a Multi-Scale Network

**DOI:** 10.3390/s23042062

**Published:** 2023-02-11

**Authors:** Nadezda Sokolova, Aiden Morrison, Knut Stanley Jacobsen

**Affiliations:** 1 SINTEF Digital, Strindveien 4, 7034 Trondheim, Norway; 2 Geodetic Institute, Norwegian Mapping Authority, Kartverksveien 21, 3511 Hønefoss, Norway

**Keywords:** GNSS, anomalous ionosphere, spatial decorrelation, short baseline, high latitudes

## Abstract

In this article, a cluster comprised of eight Continuously Operating Reference Station (CORS) receivers surrounding five supplemental test stations located on much shorter baselines is used to form a composite multi-scale network for the purpose of isolating, extracting, and analyzing ionospheric spatial gradient phenomena. The purpose of this investigation is to characterize the levels of spatial decorrelation between the stations in the cluster during the periods with increased ionospheric activity. The location of the selected receiver cluster is at the auroral zone at night-time (cluster centered at about 69.5° N, 19° E) known to frequently have increased ionospheric activity and observe smaller size of high-density irregularities. As typical CORS networks are relatively sparse, there is a possibility that spatially small-scale ionospheric delay gradients might not be observed by the network/closest receiver cluster but might affect the user, resulting in residual errors affecting system accuracy and integrity. The article presents high level statistical observations based on several hundred manually validated ionospheric spatial gradient events along with low level analysis of specific events with notable temporal/spatial characteristics.

## 1. Introduction

Of the various Global Navigation Satellite System (GNSS) error sources, radio propagation delay due to the ionosphere and its spatial variation are the most variable and serious sources of error for both the stand-alone and augmented GNSS system operations. While the ionospheric delay phenomena itself is quite well studied, accurate modelling of the spatial variation in the delay during anomalous ionospheric events and the impact of any unmodelled residuals is one of the areas that still remains a challenge. Under nominal conditions, the majority of ionospheric delay can be removed through e.g., modelling, use of multi-frequency observations, or various implementations of differential processing (a broad summary of available techniques can be found in [1]). In the case of differential processing, a small residual error can remain due to spatial and temporal decorrelation between a station pair/reference and user. The spatial decorrelation error is the largest of the two but is still very small in nominal conditions. However, during the periods of anomalous ionospheric activity, high spatial variation in the ionospheric delay can be observed [2,3,4,5] that cannot be easily mitigated, resulting in larger residual errors that can affect system accuracy and integrity [6]. While large scale slow changing variations can still be modelled with some confidence, small scale and/or rapidly moving spatial variations are more challenging to handle.

This article presents preliminary results from a 365-day long (1 April 2021–31 March 2022) monitoring campaign carried out in Northern Norway where a cluster of thirteen geodetic grade receivers centered at about 69.5° N, 19° E has been used to study the spatial decorrelation of the ionospheric delay on baseline lengths between 1.35–75 km. The location of the selected receiver cluster is in the auroral zone at night-time. The high latitude ionosphere is complex and highly variable. Multiple publications are available addressing the specific aspects of the ionospheric phenomena typical for high latitude/auroral regions such as the storm-enhanced density (SED)/tongue of ionization (TOI) creation mechanisms [7,8,9], processes behind formation and perturbations of the polar cap patches [10,11,12,13] and auroral blobs [14,15,16], as well as the general cusp dynamics associated with polar patch production [17]. Created in the dayside cusp region, the high plasma densities of the polar cap patches (polar cap patches are defined as 100–1000 km scale regions with plasma densities 2–10 times higher than that of the surrounding plasma [11,12]) move with the local magnetospheric-driven convection, typically drifting across the polar cap and becoming increasingly structured due to plasma instabilities [7,8,18]. Some of the polar cap patches exit the polar cap and enter the nightside auroral latitudes becoming boundary/auroral blobs [11,12,18] leading to strong plasma irregularities when combined with nightside aurora [13]. As discussed in [19,20], along the boundaries of such features sharp plasma density gradients with spatial scale size of several tens of kilometers exist.

Observation of small spatial-scale ionospheric delay features can be a challenge for sparse CORS networks. The inability to observe an ionospheric spatial gradient can lead to a residual ionospheric error on the user/rover side in systems employing interpolation of the distance-dependent biases to the virtual station/user location, as is the case, for example, in the Network Real Time Kinematic (NRTK) approach [21,22,23], impacting the ambiguity fixing time and success rate [6]. In addition to degrading the accuracy of the solution, such scenarios can potentially pose an integrity risk if such is intended to be supported by the system/network. Another example of systems where the inability to observe a spatial gradient can be critical, is applications such as those proposed in [24,25], where a sparse network of monitoring stations at longer baselines surrounding a local high integrity system is used to assist the real-time ionospheric gradient threat monitoring process. Spatial decorrelation of the ionospheric delay during periods with anomalous activity is a topic area studied extensively with regard to the operation of high integrity GNSS augmentations such as the Ground-Based Augmentation Systems (GBAS). Results of several studies focusing on characterization of the ionospheric gradient in different geographical regions, using the data from regional CORS networks, are available [26,27,28], as well as discussions of suitable approaches and tools for reliable monitoring and potential mitigation of the spatial variation [22,29]. These results are however limited to the observations made mainly on longer baselines (tens of kilometers), as well as the approach taken to data processing where the focus is on mapping the observed upper limits of the ionospheric spatial gradient that is to be used for defining the ionospheric threat model for a particular geographic region [30]. In many applications the distance between the user and the ground network/virtual refence station is much less than ten kilometers; therefore, spatial decorrelation of the ionospheric delay during the periods with disturbed ionosphere on shorter baselines needs further attention. Additionally, better knowledge of the local phenomena including typical spatial scale size, orientation, and propagation velocities can be of particular interest for systems where interpolation of the ionospheric residuals is carried out as well as integrity supporting systems/applications. The main focus of this article is on identification, characterization, and analysis of the observed ionospheric gradient events. The impact on the user solution and residual error characterization is on-going work to be discussed in future publications.

The remainder of this paper is organized as follows. Section 2 presents the details about the monitoring receiver cluster used for data collection and data pre-screening approach used in order to select the periods with disturbed ionosphere. Section 3 describes the data processing software and analysis steps. Statistical analysis results in terms of the observed ionospheric front slope, width, and speed, as well as example observations made on different baseline lengths, are discussed in Section 4, with conclusions drawn in Section 5 of the article.

## 2. Receiver Cluster and Data

**Figure 1:**
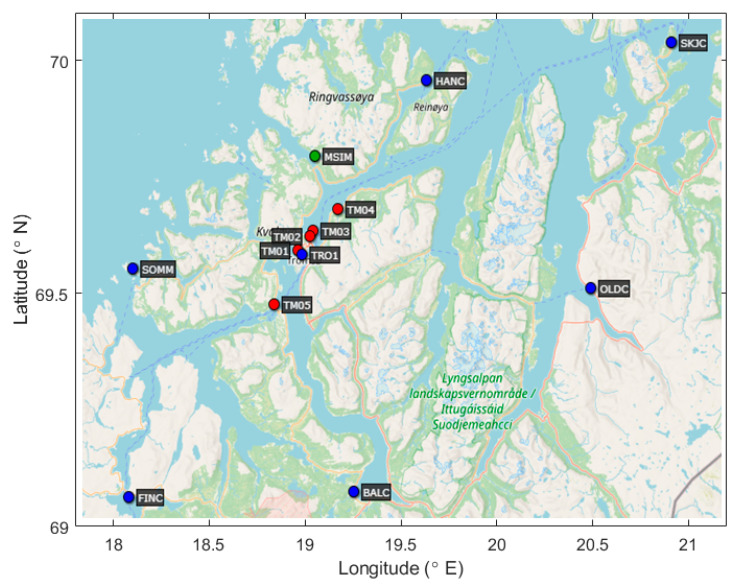
Map of the receiver locations (blue: CPOS reference receivers, green: CPOS monitoring receiver, red: test receivers).

The Norwegian Mapping Authority (NMA) operates a nation-wide NRTK positioning service “CPOS” (https://www.kartverket.no/en/on-land/posisjon/guide-to-cpos (accessed on 15 January 2023)). The service is supported by a CORS network of about 270 permanently installed receivers located across Norway. For this study, a cluster of eight CORS receivers (seven reference receivers and one monitor) covering the area around Tromsø (69.6° N, 18.9° E) were selected. These specific CORS receivers used by the CPOS service will be referred to as CPOS receivers hereafter in this article. This cluster was expanded by five additional receivers during early 2021. It is noted that these additional receivers were deployed mainly for research purposes, thus they do not serve as a part of the CPOS network. While this combination of GNSS receivers is not homogeneous, i.e., it is comprised of different geodetic grade receiver and antenna makes and models, it is believed that this fact has a negligible impact on the analysis presented in this paper. The map of the considered receiver cluster indicating receiver type (CPOS reference/CPOS monitor/test) is shown in Figure 1. An overview of station code, receiver coordinates, and receiver models used is provided in Table 1.

**Figure 2:**
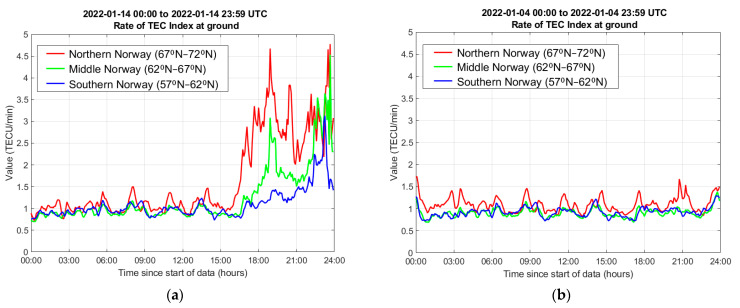
Rate of TEC Index at ground measured based on the observations from the NMA operated SATREF network. (**a**) A day with moderate-high ionospheric activity, 14 January 2022; (**b**) a day with low ionospheric activity, 4 January 2022.

For the analysis presented in this paper, a 365-day period between 1 April 2021–31 March 2022, was considered. To reduce the amount of data to be processed as well as the number of gradients to be validated manually, a re-selection process was carried out based on the Rate of TEC Index measurements [31] generated based on the observations from the NMA’s monitoring network. This pre-screening approach was selected in order to identify the days with increased ionospheric activity, as opposed to the periods with nominal behavior. NMA provides a time series of the Rate Of change of TEC Index (ROTI) for three different regions of Norway, namely its Southern (57° N–62° N), Middle (62° N–67° N), and Northern parts (67° N–72° N). As all of the receivers in the considered cluster are located beyond 67° N, the pre-selection process was based on the ROTI calculated for the Northern part of Norway. All days in the considered period for which the measured ROTI value exceeded 2 TECU/min, indicating an increase in the ionospheric activity, were used in the analysis. In total, data for 118 days was processed. Figure 2 shows examples for the ROTI measurement time series for two different days, one with a moderate to high level of activity observed and therefore used in the analysis, and the other with no notable disturbances, which was excluded.

## 3. Data Processing

To study the spatial decorrelation of the ionospheric delay for the considered cluster of receivers, the data recorded by each of the receivers was analyzed using a MATLAB-based GBAS Ionosphere Monitoring Assessment (GIMA) tool [32], the core of which was initially developed by Stanford University and is known as the Long-Term Ionosphere Anomaly Monitoring (LTIAM) [33,34]. GIMA is an evolution of the LTIAM tool carried out by EUROCONTROL in support of efficient and reliable automated ionospheric gradient detection. While the purpose of GIMA is to support analysis of larger continuous data sets without the need for preselection of days of interest, analysis of periods/days selected based on known measures of ionospheric irregularity level such as planetary K (Kp) and Disturbance, storm time (Dst) geomagnetic indexes, or Rate Of change of TEC Index (ROTI) [35,36] which is, as mentioned earlier, the approach used in this work.

Core methodology used by the GIMA tool is based on the approximation of the ionospheric spatial gradient as a moving wedge of constant, linear change in slant ionosphere delay. This geometric model approximating anomalous ionosphere behavior with simple parameters has been proposed in [26,27] based on the observation data from the CORS and WAAS reference receivers in the conterminous United States (CONUS), and adopted by numerous consecutive studies. The key parameters of this model are the gradient slope in mm/km, the width of the wedge in km, and the speed in m/sec at which the wedge moves relative to a fixed point on the ground.

In this article, only a brief description of the processing steps carried out by GIMA is provided. A detailed discussion of the structure, algorithms, and data processing approaches used can be found in [32]. The tool has been originally developed for the ionospheric gradient threat model definition in support of GBAS operation; therefore the core constellation and frequency used for the analysis are the GPS L1, as it is the basis of the currently certified GBAS concepts [37]. GPS L2 is the second frequency used by the tool, but mainly for the direct derivation of the ionospheric delay component.

First, RINEX observation data from all the considered receivers is screened for outliers and cycle slips. Following that, the slant ionospheric delay on L1 is calculated in three different ways:

(1) $$I_{\rho }=\frac{\rho_{2}-\rho_{1}}{\gamma -1},\;I_{\phi }=\frac{\phi_{2}-\phi_{1}}{\gamma -1},\;I_{CMC}=\frac{\rho_{1}-\phi_{1}}{2}$$

In (1), $I_{\rho }$ and $I_{\phi }$ are the code- and phase-based estimates, whereas the $I_{CMC}$ is the code-minus-carrier (CMC), or the code-carrier divergence estimate. $\rho_{n}$ and $\phi_{n}$ are the GNSS pseudorange and carrier phase observables for the L1 and L2 signal frequencies that can be generalized as follows:

(2) $$\begin{array}{l} \rho_{1}=r_{i}^{k}+I_{i}^{k}+\varepsilon_{\rho_{1}}\\ \phi_{1}=r_{i}^{k}-I_{i}^{k}+N_{1}+\varepsilon_{\phi_{1}}\\ \rho_{2}=r_{i}^{k}+\gamma I_{i}^{k}+c\left(IFB_{i}+\tau_{gd}^{k}\right)+\varepsilon_{\rho_{2}}\\ \phi_{2}=r_{i}^{k}-\gamma I_{i}^{k}-c\left(IFB_{i}+\tau_{gd}^{k}\right)+N_{2}+\varepsilon_{\phi_{2}}\\ \gamma =\frac{f_{1}^{2}}{f_{2}^{2}} \end{array}$$

The common term, *r*, represents the sum of the true range between the *i*th receiver and *k*th satellite, receiver/satellite clock biases, and tropospheric delays. The carrier phase observables contain an integer ambiguity, *N*, but have lower multipath and thermal noise errors than the code measurements, $\varepsilon_{\rho }\gg \varepsilon_{\phi }$. The term *I*, represents the ionospheric delay, that on the L2 frequency $\left(f_{2}\right)$ is proportional to the delay *I* on the L1 frequency $\left(f_{1}\right)$ by the squared frequency ratio γ. Additionally, hardware inter-frequency biases on the receiver, *IFB*, as well as the satellite, $\tau_{gd}$, affect the estimate of delay. The pseudorange-based estimate of the ionospheric delay, $I_{\rho }$, is noisy, but has no integer ambiguity. $I_{\phi }$ estimate is low noise, but contains integer ambiguities on both the L1 and L2. $I_{CMC}$ is the single frequency code-minus-carrier (CMC) estimate that is robust to the fragility of L2 codeless and semicodeless tracking loops, but contains both the L1 integer ambiguity and noise. The main reason for calculating all three options listed in (1) is to be able to perform consistency checks to ensure reliable detection and support the automated screening process as a large number of the detected gradients are faulty candidates caused by other processes than the anomalous ionosphere events. While not described in detail here, as part of this step a number of corrections including compensation for $\tau_{gd}$, *IFB* estimation and removal, as well as carrier-phase data levelling to remove the integer ambiguities on both frequencies are carried out.

The next step is estimation of the ionospheric spatial gradient which is carried out using the station-pair method [28]. Estimates of ionospheric slant delay are made by two reference receivers at known locations from the same GNSS satellite at the same time, and the spatial gradient is calculated simply as the difference between these two delays divided by the baseline distance between the two stations. The distances within the presumed ionospheric thin shell are deliberately not used in this approach, as the uncertainty in the thin-shell height can be high under anomalous conditions. GIMA automatically makes a record of each detected ionospheric gradient that is above a user defined threshold.

Upon event detection, carrier phase derived ionospheric delay measurements from all of the receivers in the considered cluster are used to compute the ionosphere front speed and direction. The steepest part of the ionosphere delay observed by the original pair of receivers that triggered detection is used as a reference against which the ionospheric delay measurements from all the other receivers in the cluster are compared using a pattern recognition algorithm [32] to make sure that the same front is observed by at least three of the receivers, which is the required minimum for this process. Then, the time difference between when the ionosphere front hits the reference receiver and when it hits other receivers in the cluster is computed. Finally, a set of ionosphere front speed and direction values is assumed, and for each pair of values and each receiver the difference between the measured and assumed front time is computed and the front speed and direction minimizing the sum of this difference is selected.

The last step in the process is manual validation of the detected gradients to make sure that the observed events are actually due to the ionosphere and not receiver faults or data errors.

## 4. Results and Discussion

### 4.1. Data Analysis

Data from all the monitoring receivers in the cluster (Figure 1) have been processed using GIMA for the identified 118 days. While there is no clear definition of separation between nominal and anomalous ionospheric spatial gradient, in a number of previous studies where nominal ionosphere behavior in terms of spatial gradient was addressed, it has been found that the variation is typically in the range of 0–10 mm/km [38,39] considering the GPS L1 frequency. In this study, a detection threshold equal to 50 mm/km was applied so that each of the cases for which the observed spatial ionospheric gradient exceeded 50 mm/km was considered as anomalous. This value was selected primarily to keep the number of false detections made by the GIMA automatic processing reasonable, making the manual validation a feasible task. A secondary consideration behind this threshold value selection was the expectation of a limit imposed on the minimum observable gradient due to the noise level of the carrier phase measurements.

In total 6534 events were identified for the considered period, of which 429 passed the manual validation process. First, the data was processed, and manual validation was carried out based on the RINEX data with a resolution of 30 s. Table 2 summarizes the parameter settings used for data processing in this step. Then the periods for each of the detected and validated ionospheric gradients was re-processed using a 1 s update rate for a more thorough evaluation of the event in terms of the ionospheric front slope, speed, and width. It is noted that re-processing at a 1 s rate is not a required step and has been done in this work mainly to ensure higher certainty in the anomalous gradient identification process.

One detail worth noting is that as discussed in e.g., [40], sharp plasma density gradients at the edges of the high electron density streams are frequently co-located with ionospheric scintillation. While scintillation is not the focus of the study presented in this article, it has the potential to impact or degrade the quality of the GNSS observables used for gradient estimation. In our analysis, as discussed in the previous section, the impact of scintillation is mitigated through three sequential processes including cycle slip detection and repair, false gradient rejection, and manual validation of the detected spatial gradients. This pre-screening process reduces the pool of the available observations but is considered a necessary data quantity-quality trade-off for the desired ionospheric gradient analysis.

### 4.2. Ionospheric Spatial Gradient Characterization Results

**Figure 3:**
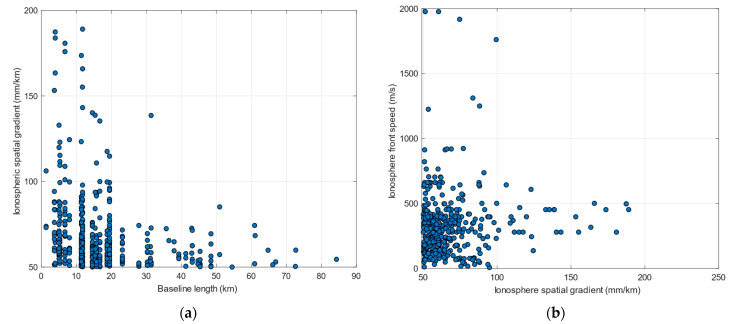
(**a**) Scatterplot of the ionospheric front slope and the baseline between monitoring stations; (**b**) Scatterplot of the ionospheric front speed and magnitude of the slope.

Figure 3a shows a scatterplot of the observed ionospheric front slope and the baseline length between the monitoring stations. As can be seen from this figure, the majority of the ionospheric gradient events as well as the highest magnitude events have been observed at the baselines ranging from 1.36–31 km. It is noted that due to higher measurement noise, in the case of the 1.36 km baseline only a single event has passed the manual validation process. Figure 3b illustrates the observed ionospheric front velocity and magnitude of the slope for each of the identified gradient events. The observed events appear to range from quasi static to moving at velocities of nearly 2000 m/s. Another important detail to note is that the ionospheric front velocity could not always be computed. As mentioned earlier, the methodology used requires that at least 3 stations have experienced and detected the same gradient. For some gradients, only a pair of stations provided a valid detection and therefore no velocity can be computed.

**Figure 4:**
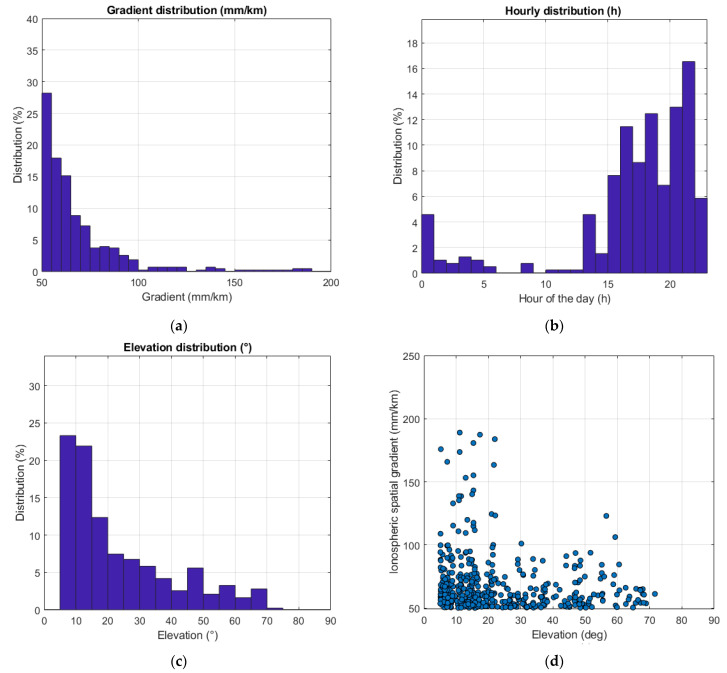

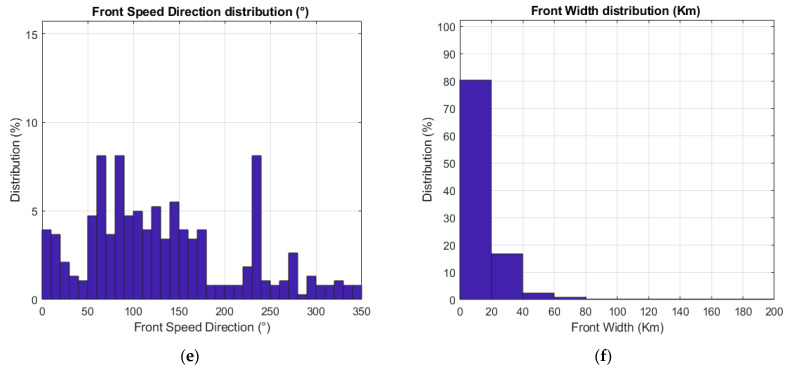
(**a**) Distribution of the ionospheric spatial gradient observations.; (**b**) Ionospheric spatial gradient distribution per hour of day (local time); (**c**) Ionospheric spatial gradient distribution as a function of satellite elevation; (**d**) Scatterplot of the ionospheric spatial gradient and satellite elevation; (**e**) Ionosphere front direction of motion distribution; (**f**) Ionospheric front width distribution.

Figure 4a shows the distribution of the observed ionospheric gradient events. As one can see, the vast majority of the events are under 100 mm/km. The highest observed gradient is 189 mm/km, detected between the TM01 and TM05 stations separated by 11.76 km. As the solar activity was not at its maximum during the time period considered in this study, larger gradients might be expected when the solar activity is higher. Figure 4b illustrates the gradient distribution as a function of time/hour of day. Most of the events occurred during evening and nighttime. This can be explained by the average location of the auroral oval, which at night is centered over northern Scandinavia. This is also commensurate with the observations made in [41,42], both in terms of ROTI and ionospheric scintillation. Figure 4c,d illustrate the relationship of the distribution and the magnitude of the observed gradients with the satellite elevation angle. More than 45% of the events were observed on elevation angles below 15°. The absence of events on elevation angles above 85° can be explained by the absence of measurements due to satellite geometry on site. Most of the largest gradients were also observed at lower elevation angles ranging from 5° to 20°. Figure 4e shows the distribution of the observed gradients as a function of the ionosphere wave front direction of motion relative to the ground, whereas the distribution of the front width observations is shown in Figure 4f. The vast majority of the gradient width (or the scale size) estimates are below 20 km, indicating that most of the observations are small spatial scale events.

**Figure 5:**
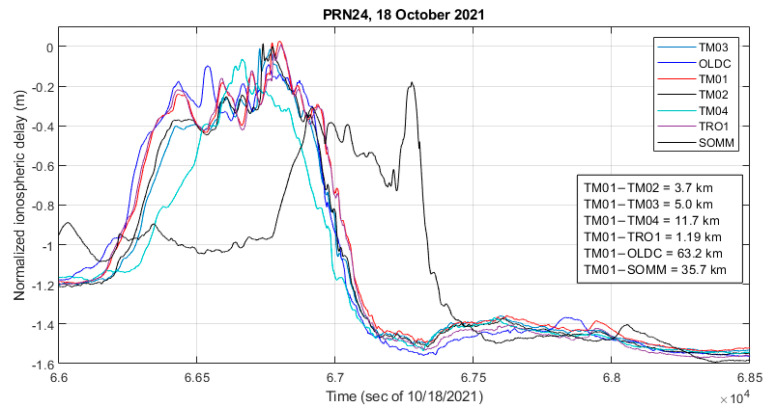
Variation in the slant ionospheric delay as observed on GPS PRN24 by a subset of monitoring stations. Elevation angle of 48°–60° across stations during the plotted time period. Estimated propagation speed over ground of 155 m/s.

Figure 5 shows an example of the observed slant ionospheric delay variation. It should be noted that in this figure the normalized delay values are shown, thus only the variations of these delays should be examined.

The subset of receivers is selected to visually emphasize the characteristics of the event. The rapid change in delay associated with both the leading and falling edges of the enhanced ionospheric delay feature took just a few minutes. In the case of the falling edge for example the decrease of about 1.2 m took 3.7 min. Observation of such spatially small-scale structures can be a challenge for larger scale networks. When comparing the results for individual stations in Figure 5, one can see that the underlying primary trend in the normalized traces is quite similar, but that each station experiences differences in the smaller scale and higher frequency ionospheric residuals. While the feature was propagating along the cluster in the North-West direction, the slopes on both the rising and falling edges changed, as can be observed in the cases of the TM04 and SOMM stations. When considering the longest slope at the falling edge of the feature, and using the TM01 station as a reference, correlation between the slopes observed by the TM01 and TM04 stations is 0.79, correlation between TM01 and SOMM is 0.74, while correlation between TM01 and the rest of the stations considered in Figure 5 is between 0.98 and 0.99.

**Figure 6:**
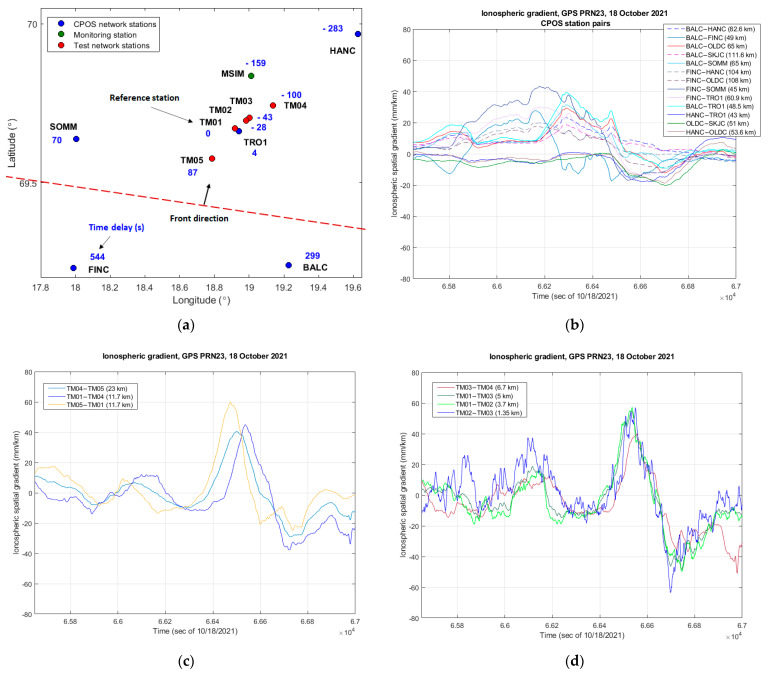
(**a**) Observed time delay relative to the reference station (here TM01); (**b**) Ionospheric spatial gradient observed by the CPOS station pairs in the cluster, baselines ranging from 43–111 km; (**c**) Ionosphere spatial gradient observed by the test receivers located on baselines ranging from 11.7–23 km; (**d**) Ionospheric spatial gradient observed by the test receivers located on the shortest baselines ranging from 1.35–6.7 km.

Figure 6 shows an example case of a smaller enhanced ionospheric delay feature where the spatial delay gradient observed by the receivers located on shorter baselines was higher than the observations made by the CPOS receivers.

In this example, the ionospheric front was approaching the receiver cluster at 254 m/s from the South-West direction as shown in Figure 6a, indicating the observed time offsets with regard to the reference station. It is noted that in Figure 6a only the stations for which the time offset could be determined are shown. As shown in Figure 6b, the maximum observed peak-to-peak ionospheric spatial delay gradient value considering only the CPOS stations was about 56 mm/km within the plotted period (HANC-TRO1 station pair). Figure 6c,d show the same event as observed by stations located on shorter baselines, where noticeably higher peak-to-peak variation was detected. In particular, 105 mm/km considering the TM01–TM02 station pair, and 119 mm/km as observed considering TM02–TM03 with the caveat that this is a noisier trace given the shortest baseline length. While the variation between the observations shown in Figure 6c,d is relatively small, it is clearly visible that noise becomes a non-negligible component of the observations made on shorter baselines.

**Figure 7:**
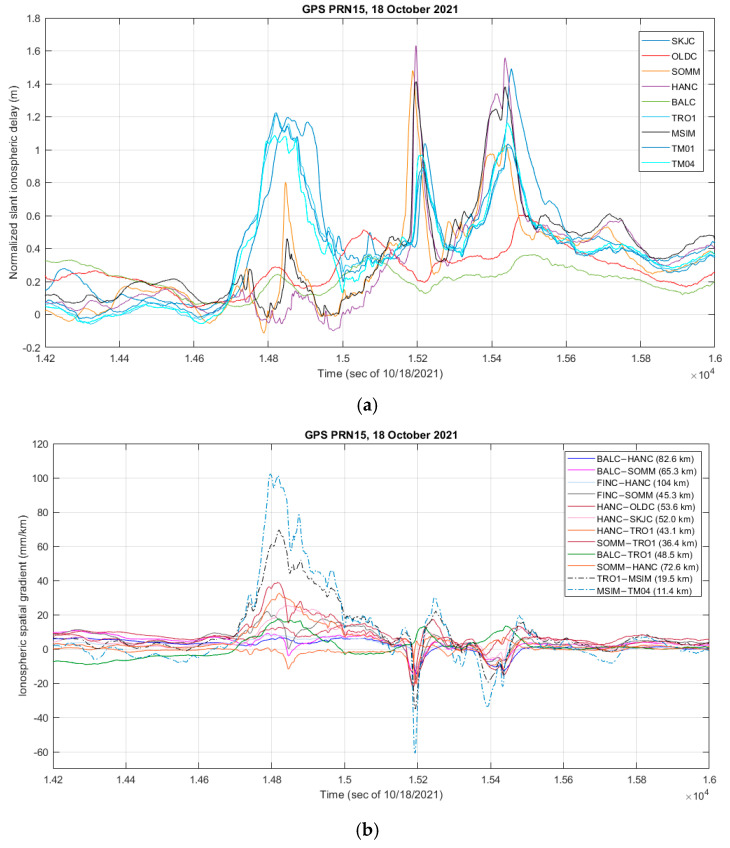

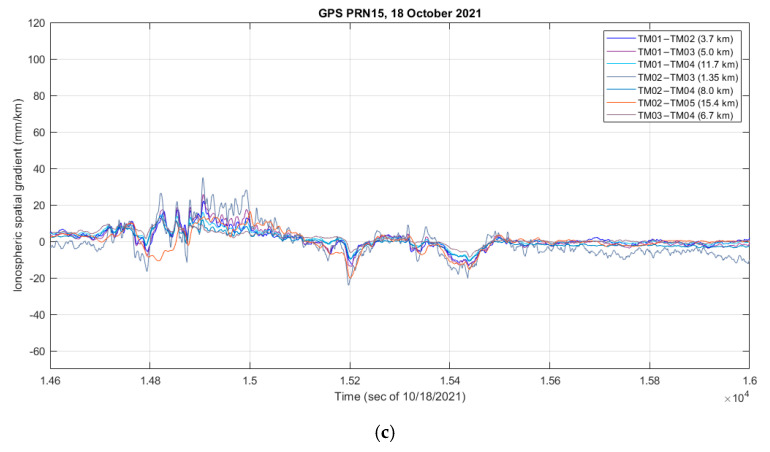
(**a**) Variation in the slant ionospheric delay as observed on GPS PRN15 by a subset of monitoring stations. Elevation angle of 28°–30° across stations during the plotted time period; (**b**) Ionospheric spatial gradient values. Higher values on MSIM-TRO1 and MSIM-TM04 station pairs indicate spatial variation in the ionospheric gradient between the higher North-West segment and the rest of the cluster; (**c**) Ionospheric spatial gradient values observed by the test receiver subset.

Another example of a relatively steep but small scale and spatially isolated event is shown in Figure 7a–c, where an ionospheric front was detected to be approaching the receiver cluster from the South-East. As can be observed from Figure 7a, showing the normalized slant ionospheric delay for a subset of receivers, some stations observed the entirety of the event (SKJC, TRO1, TM01, and TM04) while a second category observed almost no activity (OLDC and BALC), and a third category observed a much smaller and shorter duration though still notable increase in the ionospheric delay (SOMM, HANC, and MSIM). In this case, while there was a larger spatial gradient observed by the MSIM-TRO1 and MSIM-TM04 station pairs, as shown in Figure 7b with dashed lines, no gradient was observed on either the test receiver pairs, as shown in Figure 7c, or the rest of the CPOS receiver pairs, as illustrated in Figure 7b by solid line traces.

## 5. Conclusions and Future Work

Results based on the analysis of 118 days with increased ionospheric activity have been presented and discussed. Within this term, a total collection of 6534 candidate ionospheric spatial gradient events was detected by the GIMA analysis tool, out of which 429 passed manual validation and were further considered here. It is noted that GIMA has an option to apply a neural network for filtering out false gradients which has not been applied in this particular study but is planned to be used for later analysis where longer periods of data will be analyzed. The results of this study will be used in part to help train and validate the neural network model used in GIMA so that the high level of manual effort required to extract the data in this study is not without purpose.

Both high level aggregate statistics over the full term of the events, as well as low level analysis of specific events demonstrating notable data features, have been produced, presented, and, discussed. Of the validated 429 events, the scale size and the layout of the various network stations allowed us to conclude that more than 95 percent of the identified gradient events seen in this region comprised spatially small-scale events with estimated widths of 40 km or less. The vast majority of the observed ionospheric gradient values were under 100 mm/km. This type of event is necessarily rare and difficult to observe with large scale networks as the isolated regions of disturbance can pass undetected between station pairs. This specific behavior was demonstrated within the elaborated events that showed clearly where the activity could be seen only with subsets of the local receiver cluster.

Such events, while difficult to detect, still pose a potential integrity issue to user solutions if interpolation of the atmospheric residuals is done based on a larger scale network/cluster, and are unlikely to be mitigated by approaches which rely on feature matching/correlation as they are simply not observed or have a significantly different slope/magnitude/duration signature at a subset of network stations.

## Figures and Tables

**Table 1:** List of the receivers included in the cluster indicating location and receiver model.

Station Code	Latitude (° N)	Longitude (° E)	Receiver Model
TM01	69.670	18.918	Leica GR50
TM02	69.695	18.982	Leica GR50
TM03	69.705	19.001	Leica GR50
TM04	69.743	19.136	Leica GR50
TM05	69.576	18.784	Leica GR50
BALC	69.240	19.226	Trimble NetR9
FINC	69.231	17.987	Trimble NetR9
HANC	69.968	19.626	Trimble NetR9
OLDC	69.604	20.534	Trimble NetR9
SKJC	70.034	20.976	Trimble NetR9
SOMM	69.637	18.004	Trimble NetR9
TRO1	69.663	18.939	Trimble NetR9
MSIM	69.835	19.011	TPS NET-G5

**Table 2:** Summary of the basic GIMA parameter settings applied for data processing.

Sampling rate (prior manual validation)	30 s for initial processing
Elevation mask	5°
Ionosphere height	350 km above the earth surface
Ionospheric gradient detection threshold	50 mm/km
Minimum number of points above threshold to declare detection	1
Ionospheric front speed computation:	
Minimum speed Maximum speed Step	20 m/s 2000 m/s 5 m/s
Distance between stations (min, max)	min: 1 km; max: 150 km

## Data Availability

The Rate of TEC Index (ROTI) observations based on the Norwegian Mapping Authority operated network of receivers can be obtained at https://sesolstorm.kartverket.no/download.xhtml.
